# Recalling Life’s Highs and Lows in Older Age: Hierarchical Clustering Methods to Topics, Themes, and Structures

**DOI:** 10.1093/geroni/igaf122.3406

**Published:** 2025-12-31

**Authors:** Hsiao-Wen Liao, Nikhil Shanbhogue, Hongchen Wu, Thomas Oltmanns

**Affiliations:** Georgia Institute of Technology, Atlanta, Georgia, United States; Georgia Institute of Technology, Atlanta, Georgia, United States; Georgia Institute of Technology, Atlanta, Georgia, United States; Washington University in St. Louis, St. Louis, Missouri, United States

## Abstract

Autobiographical memory becomes richer and more complex, forming a life story, as people develop and grow. This study applies BERTopic—a transformer-based modeling method that leverages BERT-based sentence embeddings and hierarchical clustering—to identify interrelated topics and themes in older adults’ life story events of high and low points. Participants, part of the St. Louis Personality and Aging Network study (N = 468), completed a life story interview. Memories were transcribed verbatim for text analysis. Central analyses involved transforming the text into numerical representations by generating embeddings, reducing dimensions following cluster analysis, and extracting keywords for topic representation. Ward’s method was used to inform hierarchical dendrograms, through which broader themes and memory structures were inferred. Results indicated that older adults’ high points contained more topics and were less semantically similar (k = 8; cosine similarity ranging from 0.63 to 0.87) than their low points (k = 4; cosine similarity ranging from 0.74 to 0.9). Three distantly related (i.e., family joy, career milestones, religious fulfillment) and two tightly clustered themes (i.e., illnesses and loss, marital conflicts) respectively emerged from the topics in high and low points. This study sheds new light on applications of natural language processing techniques to the mental representations of key elements and links in life stories. Findings suggested that older adults’ high points encompassed relatively loosely interconnected experiences across multiple facets of life. Experiences in low points were more thematically alike. In discussing these findings, we draw on the life story schema theory and research on autobiographical memory development.

